# Large magnetoelectric effects mediated by electric-field-driven nanoscale phase transformations in sputtered (nanoparticulate) and electrochemically dealloyed (nanoporous) Fe–Cu films†
†Electronic supplementary information (ESI) available: Calculation for estimating porosity. The expanded graph of data in Fig. 3, which includes intermediate 0 V steps. The oxygen XPS data. See DOI: 10.1039/c8nr03924k


**DOI:** 10.1039/c8nr03924k

**Published:** 2018-07-13

**Authors:** Shauna Robbennolt, Alberto Quintana, Eva Pellicer, Jordi Sort

**Affiliations:** a Departament de Física , Universitat Autònoma de Barcelona , E-08193 Bellaterra , Spain . Email: shaunaaryn.robbennolt@uab.cat ; Email: jordi.sort@uab.cat; b Institució Catalana de Recerca i Estudis Avançats (ICREA) , Pg. Lluís Companys 23 , E-08010 Barcelona , Spain

## Abstract

Large magnetoelectric effects are observed in as-sputtered (nanoparticulate-like) and electrochemically dealloyed (nanoporous) 200 nm thick Fe–Cu films. Application of positive voltages decreases both the saturation magnetization (*M*_S_) and coercivity (*H*_C_) of the films, while negative voltages cause the reverse effect (increase of *M*_S_ and *H*_C_). The relative variations are as high as 20% for *M*_S_ and beyond 100% for *H*_C_, both for the as-sputtered and dealloyed states. These changes in magnetic properties are caused by controlled and reversible electric-field-driven nanoscale phase transformations between face-centered cubic (*fcc*) and body-centered cubic (*bcc*) structures. These phase transitions are in turn due to selective redox reactions induced by the applied voltage, which can be regarded as a “magnetoionic effect.” The controlled tuning of *H*_C_ and *M*_S_ with the moderate values of applied voltage, together with the sustainable composition of the investigated alloys (not containing noble metals, as opposed to many previous works on magnetoelectric effects in thin films), pave the way towards the implementation of magnetic and spintronic devices with enhanced energy efficiency and functionalities.

## Abstract 2

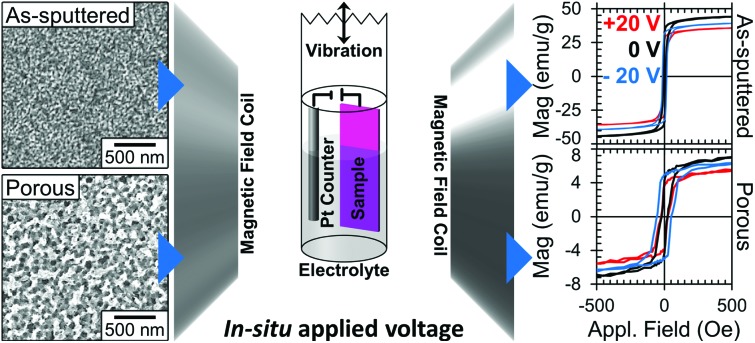
Large magnetoelectric effects are observed in as-sputtered (nanoparticulate-like) and electrochemically dealloyed (nanoporous) 200 nm thick Fe–Cu films.

## Introduction

Voltage control of magnetism is an exciting area of research due to the increasing demand to create smaller and more energy-efficient spintronic devices. These devices aim to use the spin state and associated magnetic moment to store and manipulate information in addition to electric charge, which is widely utilized in microelectronics.^1^–^4^ Conventional magnetic storage devices use magnetic fields in order to write information, but as the memory element size gets smaller, creating a sufficiently high and locally confined magnetic field becomes increasingly more challenging. Unfortunately, interference between the magnetic field and neighbouring memory elements is often unavoidable. In addition, the electric currents required to create such magnetic fields bring about dissipation of energy in the form of heat due to the Joule heating effect. One method to overcome the need for external magnetic fields is to use spin-polarized currents to write the information (spin-torque effect). While successful, generation of spin-polarized currents is also energetically costly. The use of an applied voltage rather than magnetic fields or spin-polarized currents would have the potential to significantly decrease power consumption.^1^–^5^

There are a number of methods that have been proposed to modify the magnetic properties of materials with the application of voltage. One option would be the use of single-phase multiferroic materials (MFs) which have both ferroelectric and ferromagnetic (or antiferromagnetic) ordering. However, there are very few intrinsic MFs with room temperature magnetization and those that do exist often have low magnetization and weak magnetoelectric coupling.^4^,^6^,^7^ An alternative to intrinsic MFs are “composite MFs,” which mechanically strain-couple piezoelectric and magnetostrictive materials. While useful in a wide range of other applications, composite MFs have remained unsuitable for spintronic devices because of issues such as clamping with the substrate (which strongly limits the generated strain) or mechanical fatigue.^4^,^5^,^8^

Aside from MFs, direct application of voltage to ultrathin metallic films has been also shown to be successful to tune magnetic properties to some extent.^9^,^10^ The challenge with ultrathin film-based devices is that as element sizes decrease, the superparamagnetic limit is approached, so in order to have stable magnetization, a fairly large area element is required. There is also the drawback of surface passivation, which can be prominent in ultrathin films.

Another promising route for voltage control of magnetization is through magnetoionic manipulation.^11^–^18^ Here, an applied voltage is used to induce the migration of ions, most commonly O^2–^ ions, in a stack containing a metallic magnetic layer next to a metal oxide layer. The applied electric field can be used to move the O^2–^ ions from the metal oxide layer to oxidize part of the metallic magnetic film causing a change in magnetism. A reverse electric field can then be used to move the ions back into the metal oxide layer, thereby reducing the magnetic film back to a metallic state and recovering the previous magnetic properties. This process is an interfacial effect and is therefore most relevant for architectures that have large interfacial areas, such as ultrathin films. Magnetoionic manipulation can also be applied to other high surface area structures, such as nanoporous films or even sputtered thin films, as presented here. The primary disadvantages to magnetoionic manipulation of magnetism are that the process is slow and it often needs to be thermally assisted. Furthermore, magnetoionic devices typically consist of specific bilayer structures that contain a magnetic metal in contact with layers that are highly prone to release or accept oxygen depending on the electric field polarity (*e.g.* Gd_2_O_3_, HfO_2_).^11^,^12^,^14^

Nanoporous, metallic thin films are an attractive candidate for voltage control of magnetism because they have very high surface areas and the ability to have more magnetic material per unit of area than ultrathin films.^19^–^27^ The two most common methods used to create nanoporous, metallic materials are the use of a sacrificial template,^22^,^28^–^31^ such as micellar polymer or silica beads, or selective corrosion,^19^–^21^,^23^–^25^,^32^,^33^ including chemical and electrochemical dealloying. Here, we utilize electrochemical dealloying in order to induce nanoporosity in sputtered thin films. Electrochemical dealloying is a method that is widely used to fabricate nanoporous metals. A metal alloy is placed in an electrolytic solution and a voltage is applied which causes the dissolution of the more electrochemically active element leaving a nanoporous structure primarily composed of the less electrochemically active element. While this method had been primarily applied to bulk metals,^19^,^21^,^23^–^25^,^32^,^34^–^36^ we have recently reported on the use of electrochemical dealloying to induce nanoporosity in ribbons and thin films of Fe–Cu.^19^,^37^

Fe–Cu is an interesting material system due to its favourable magnetic properties and its suitability for use in sustainable devices. Fe is an Earth-abundant element and both Fe and Cu are relatively cheap which makes Fe–Cu an attractive alternative to noble metal-based material systems like those often used for electrochemical dealloying which contain Au, Ag and/or Pt.^21^,^24^,^34^,^38^,^39^ Furthermore, Fe and Cu are both non-toxic, making Fe–Cu a potential alternative to Co-based material systems as well. Fe–Cu is a soft magnetic material and its magnetic properties are highly tuneable with composition. In terms of structure, Fe alone is natively body-centered cubic (*bcc*) and Cu is natively face-centered cubic (*fcc*). Kuch *et al.* did theoretical studies which predicted that Fe can be induced into an *fcc* structure in Fe–Cu alloys and that in this structure, Fe can have a higher magnetic moment per Fe atom than in the *bcc* structure.^40^ Furthermore, Huang *et al.* found experimentally that alloys with high Fe content, up to 80 at%, could still crystallize forming a stable *fcc* structure.^41^

Here, we investigate the effects of an *in situ* applied voltage on the magnetic properties of nanoparticulate and nanoporous Fe–Cu thin films. The nanoparticulate films are prepared by co-sputtering Fe and Cu onto Si substrates. Then, electrochemical dealloying is used to induce nanoporosity. The magnetic properties of both the as-sputtered and dealloyed films are investigated as a function of applied voltage. In order to apply the voltage, an electric double layer setup is used in which the film is submersed in an anhydrous electrolytic solution and a voltage is applied between the film and a Pt counter electrode. Applying a positive voltage is found to decrease both saturation magnetization (*M*_S_) and coercivity (*H*_C_) while negative voltages increase both parameters. The crystal structure is probed by X-ray diffraction (XRD) and the surface oxidation is investigated using X-ray photoelectron spectroscopy (XPS). Interestingly, the ratio of *fcc* to *bcc* structure in the films as well as the surface oxidation are found to change as a function of applied voltage. We believe that these structural transformations are mainly responsible for the observed changes in the magnetic properties, which can thus be considered as magnetoionically driven to some extent.

## Experimental

### Preparation of sputtered films

Thin films were prepared by co-sputtering Fe and Cu at room temperature in an AJA International, Inc. magnetron sputtering system. Clean Si substrates (5 × 10 mm^2^) were mounted and then the chamber was put under vacuum to achieve a pressure ∼10^–7^ torr. First, a layer of Cu was deposited over the entire substrate by sputtering Cu at 100 W (RF) for 5 min. A small section at the end of the substrate (5 × 2 mm^2^) was then protected before the Fe–Cu layer was deposited by co-sputtering Fe at a power of 200 W (DC) and Cu at a power of 80 W (RF) for 15 min resulting in thin films with thicknesses of about 200 nm. The section of the substrate with Cu only was subsequently used to connect the leads for voltage application in both the electrochemical dealloying process and during the magnetoelectric measurements.

### Electrochemical treatment to create nanoporosity

The films were treated using an Autolab PGSTAT302N potentiostat/galvanostat in a 3-electrode configuration. The electrolytic solution was 40 mM nitric acid in water. The sputtered films were used as the working electrode with the entire Fe–Cu film submerged in the electrolyte. The counter electrode was a platinum spiral and the reference electrode was Ag/AgCl (3 M KCl) with 20 mM nitric acid as the exterior solution. The films were dealloyed at +0.5 V *vs.* Ag/AgCl for 60 s, they were subsequently quickly removed from the solution and finally rinsed with distilled water and dried. The entire procedure was conducted in air.

### Magnetic measurements with applied voltage

**Scheme 1:**
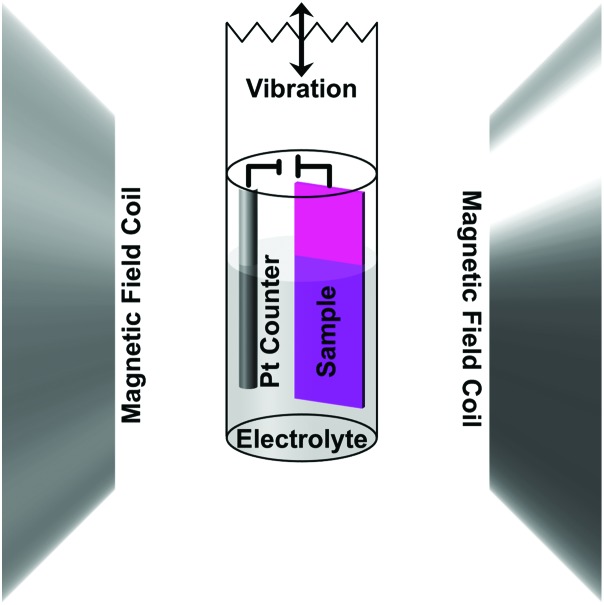
A schematic drawing of the experimental setup for applying an electric field *in situ* while measuring magnetic properties in a vibrating sample magnetometer (VSM). The sample was used as the working electrode with a Pt counter electrode, which were both submersed in an electrolyte solution made of sodium and hydroxyl ions dissolved in propylene carbonate. The small cylindrical cell was mounted to a VSM sample holder and placed in the instrument – the magnetic field coils shown here. The vibration direction, indicated on top, was along the axis of the sample holder. The sample was measured in-plane.

The magnetic properties were measured using a MicroSense (LOT-QuantumDesign) Vibrating Sample Magnetometer (VSM). The voltage was applied using an Agilent B2902A power supply. A custom electrochemical cell was constructed to be held at the end of a VSM sample holder (Scheme 1 shows a drawing of the cell setup). The small cylindrical cell was filled with propylene carbonate (anhydrous from Sigma-Aldrich [310328] 99.7%, 0.002% or 20 ppm H_2_O) previously treated with metallic sodium to remove any traces of water. As a result, the propylene carbonate solution contained a small amount of Na^+^ and OH^–^ ions (5 ppm or 0.2 mM Na^+^ as determined by ICP analysis). A two-electrode system was used in which the sample was the working electrode and a Pt wire was used as the counter electrode. Hysteresis loops were measured as a function of applied voltage in an in-plane configuration. Prior to each measurement, the voltage was set and held without measurement for 40 min to let the system stabilize. Measurements were done on as-sputtered and nanoporous films made from electrochemically dealloying the sputtered films. The voltage was applied during the measurements, which took 20 min, making the total time a given voltage was applied 1 h. The initial state (0 V) was measured first and then a low positive voltage (+10 V) was applied followed by another 0 V measurement before a low negative voltage (–10 V) was applied. This process was then repeated for higher voltages (±20 V), hence making the entire progression 0 V, +10 V, 0 V, –10 V, 0 V, +20 V, 0 V, –20 V, 0 V. Note that due to the small thickness of the electric double layer created at the interface between the sample and propylene carbonate (typically around 0.5 nm), electric fields of the order of tens of MV cm^–1^ are created at the surface of the alloy for the mentioned values of applied voltages.

### Structural & elemental characterization

Field Emission Scanning Electron Microscope (FE-SEM, Zeiss Merlin) with Energy Dispersive X-ray Spectroscopy (EDX) capabilities was used to characterize the microstructure of the films and the elemental composition. XRD patterns were collected on a Philips X'Pert diffractometer using Cu K_α_ radiation. The XRD patterns for ±20 V were collected by applying the given voltage for 1 h and then removing the voltage to do the XRD measurement. XPS analyses were performed using a PHI 5500 Multitechnique System (Physical Electronics) with a monochromatic Al K_α_ source under ultra-high vacuum. All XPS scans were carried out on the film surface. The XPS measurements for the various voltages were done *ex situ*, after having applied the voltage for 40 min before doing the XPS measurement. The sample magnetization of the VSM data was normalized by mass as determined by Inductively Coupled Plasma (ICP) Spectroscopy. The ICP was done on an ICPE 9000 from Shimadzu by dissolving each sample in 3 mL in a 1 : 1 solution of water and nitric acid (68%).

## Results and discussion

**Fig. 1:**
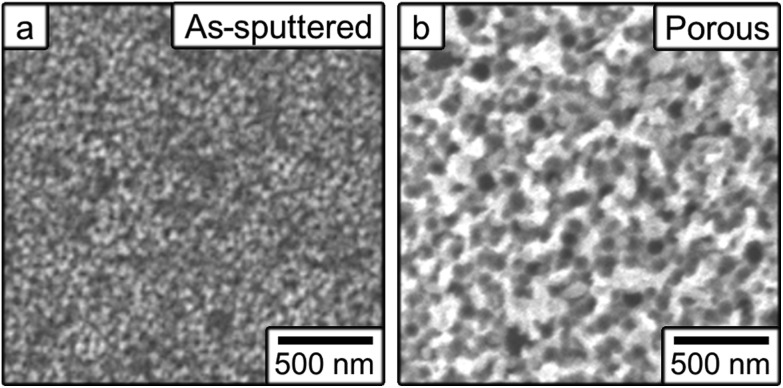
Top-view scanning electron microscopy images of (a) the as-sputtered film and (b) the film made nanoporous by electrochemical dealloying.

Fig. 1 shows top-view SEM images of the as-sputtered (a) and the films prepared by electrochemical dealloying (b). The as-sputtered films exhibit a nanoparticulate morphology, whereas clear nanoporosity is induced during the electrochemical treatments. In both cases, the surface area is higher than it would be for smooth, fully dense films. The elemental composition (atomic percent) was determined from EDX analyses to be Fe_58_Cu_42_ for the as-sputtered films and Fe_34_Cu_66_ for the dealloyed films. The difference in elemental compositions is due to the electrochemical dealloying process that causes the Fe to be preferentially dissolved. The porosity was estimated to be approximately 16% in the as-sputtered film, believed to be due to in-grain porosity, and 58% in the porous film. These values were calculated using *d*-spacing values from XRD and the masses determined by ICP and the calculation is shown in the ESI.†

**Fig. 2:**
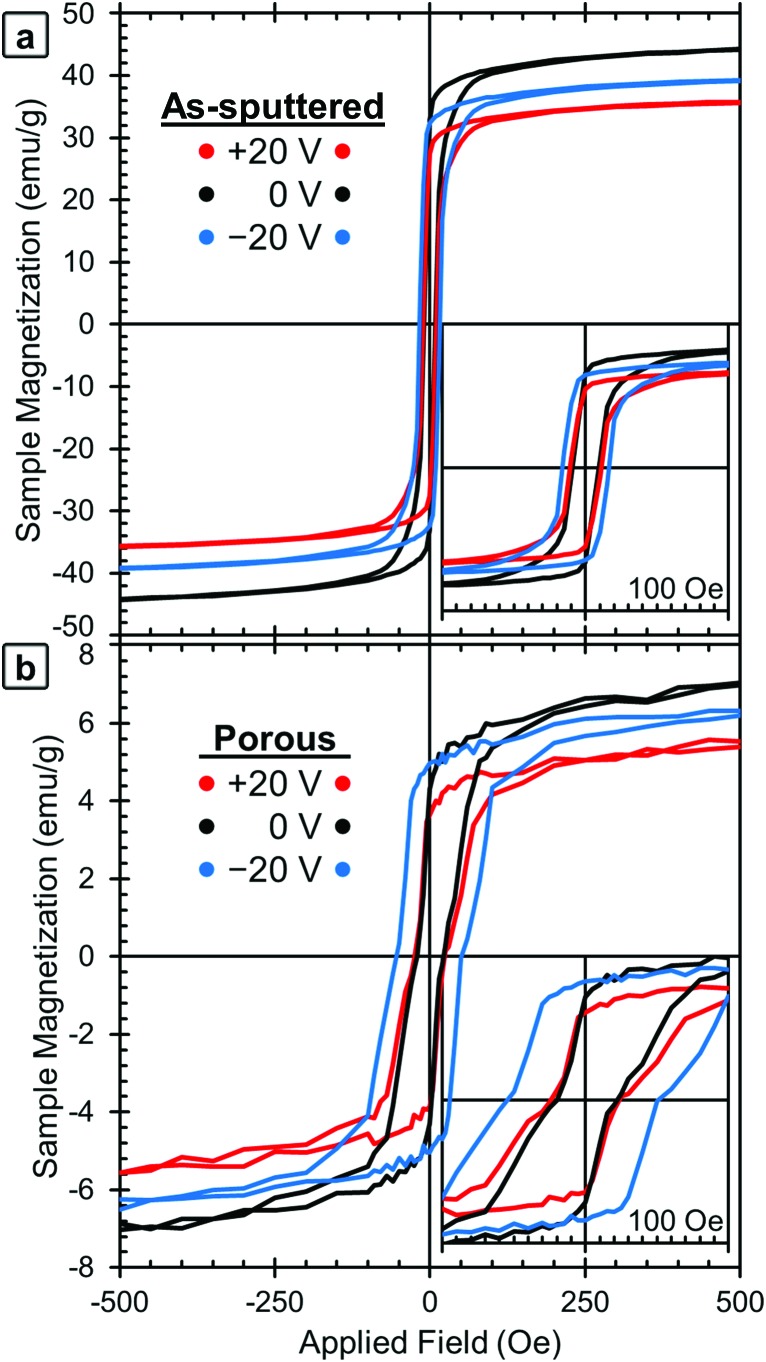
Room-temperature magnetic hysteresis loops of (a) the as-sputtered film and (b) the porous film (after dealloying). The initial state (0 V) is shown in black, together with the loops obtained applying +20 V (in red) and –20 V (in blue). For clarity, the ±10 V curves were omitted, but that data is included in Fig. 3.

The magnetic properties were measured at room temperature using VSM (Fig. 2). The voltage was applied using an electrochemical cell in which propylene carbonate with dissolved Na^+^ and OH^–^ ions (0.2 mM Na^+^ as determined by ICP analysis) was used as the electrolytic solution. As described above, in order to apply voltage *in situ*, the film and a Pt counter electrode were mounted in a specially made cylindrical cell, which was filled with the electrolytic solution and mounted to a VSM sample holder (see Experimental section).

*M*–*H* loops of the samples in their initial states were measured first (shown in black lines, labeled 0 V, in Fig. 2a for the as-sputtered film and 2b for the dealloyed film). The as-sputtered film is found to have a higher *M*_S_ and lower *H*_C_ than the porous film. These differences are due to the porosity itself and the change in composition caused by the dealloying process. The difference in *M*_S_ is attributed to the difference in composition (*i.e.*, the as-sputtered film has more Fe, Fe_58_Cu_42_*vs.* Fe_34_Cu_66_, and therefore a higher *M*_S_). However, the difference in coercivity is contrary to the expectation that the as-sputtered film should have a higher *H*_C_ since it contains more Fe. The increase of *H*_C_ after dealloying is attributed to the nanoporosity, since the reduced lateral dimensions of the pore walls may inhibit domain wall propagation. This is consistent with previously published results.^37^,^42^

**Fig. 3:**
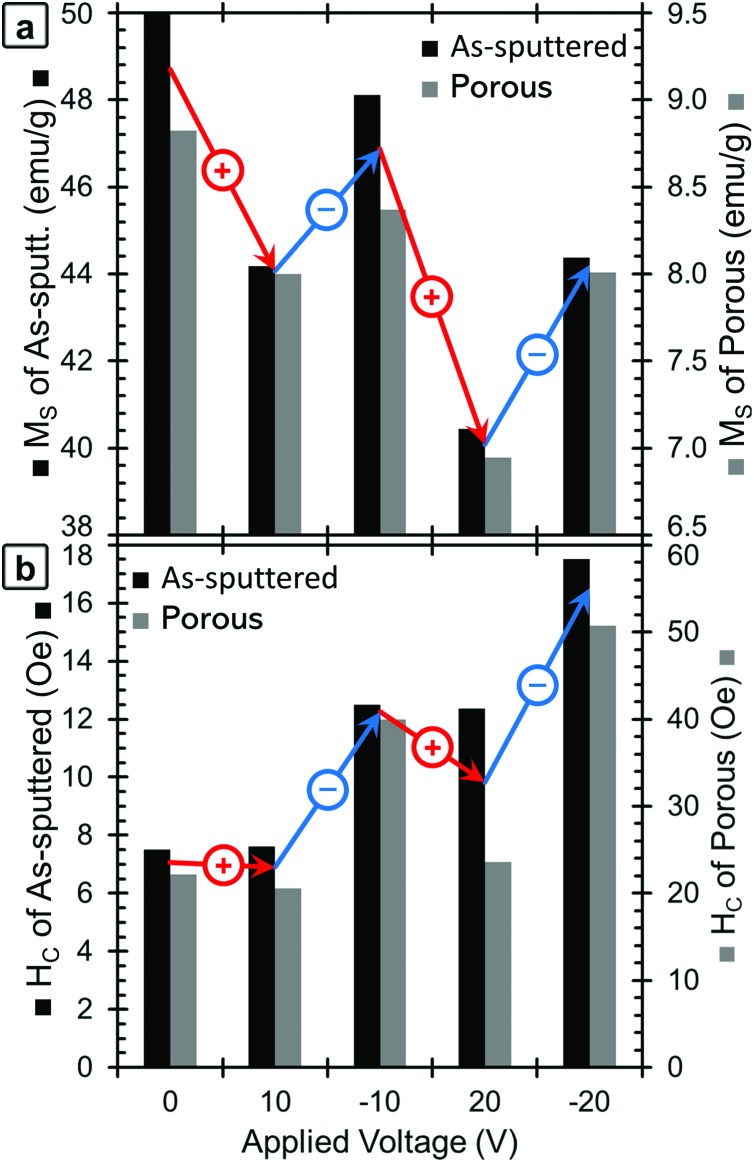
A graphical presentation of the dependence of (a) *M*_S_ and (b) *H*_C_ on the applied voltage for the as-sputtered (black, left axis) and nanoporous (grey, right axis) samples. The voltages are presented in chronological order. For clarity, red arrows show the changes when positive voltages are applied, and blue arrows indicate changes when negative voltages are applied. Note that the values of *M*_S_ correspond to the maximum applied field (20 kOe); hence they are higher than the values of *M* at 500 Oe from Fig. 2.

The red lines in Fig. 2 show the *M*–*H* loops of the samples at +20 V and the blue lines show the *M*–*H* loops taken with –20 V applied. The chronology of the voltage application is important to take into account (see Experimental section). The positive voltage causes the *M*_S_ values to decrease and then *M*_S_ is only partially recovered during the application of the negative voltage, which is the reason why the 0 V curves show the highest *M*_S_ values. The *M*_S_ and *H*_C_ of both the as-sputtered (black) and porous (grey) samples are presented as a function of applied voltage in Fig. 3a (*M*_S_) and 3b (*H*_C_). Here, the voltage sequence is shown as chronologically applied (as indicated by the arrows). During the testing, a measurement was done at 0 V between each of the voltages presented (as described above). The results of the 0 V measurements (aside from the initial one) are omitted here for clarity, but can be found in Fig. S1.† As a guide to the eye, Fig. 3 is marked with red arrows to indicate when positive voltage is applied and blue arrows indicate when a negative voltage is applied. Looking first at the trend in *M*_S_ (Fig. 3a), in the initial state, the as-sputtered film has an *M*_S_ of 50.0 emu g^–1^ and the porous film exhibits an *M*_S_ of 8.8 emu g^–1^. When +10 V is applied, *M*_S_ decreases for both the as-sputtered and nanoporous films.

When –10 V is applied, *M*_S_ increases to some extent, but does not fully recover to the initial values. For a larger positive voltage (+20 V), *M*_S_ again decreases reaching the lowest values of 40.4 emu g^–1^ for the as-sputtered film and 6.9 emu g^–1^ for the porous film. Finally, when –20 V is applied, *M*_S_ again increases, but does not fully recover back to the value at –10 V. The saturation field (*H*_S_) seems to show a similar trend and is found to decrease upon the application of positive voltages and increase upon the application of negative voltages. However, it should be noted that there is a relatively high amount of error in determining *H*_S_ from this data due to the noise level, so the exact values are not included here.

Looking next at the trend in *H*_C_ (Fig. 3b), in the initial state, the as-sputtered film has an *H*_C_ of 7.5 Oe and the porous film has an *H*_C_ of 22 Oe. When +10 V is applied, the *H*_C_ of the as-sputtered film remains unchanged while a slight decrease is observed for the porous (dealloyed) film. In contrast, when –10 V is applied, *H*_C_ increases to above the initial values. When a large positive voltage (+20 V) is applied the *H*_C_ of the as-sputtered film decreases very slightly while the *H*_C_ of the porous film decreases almost back to its initial value. At –20 V, the *H*_C_ again increases, reaching the highest values of 17.5 Oe for the as-sputtered film and 51 Oe for the porous film. This represents an increase of more than 100% with respect to *H*_C_ in the films before voltage is applied. Importantly, such an increase is much higher than the previous voltage-driven coercivity modulation reported for nanoporous Cu–Ni films using the same electrolyte.^46^

**Fig. 4:**
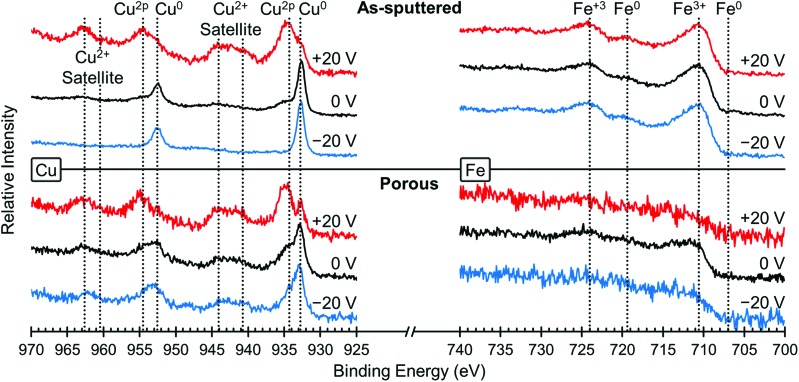
XPS elemental spectra of Cu (left) and Fe (right) for the as-sputtered film (top) and the nanoporous film (bottom). The initial state (0 V) is shown in black, while the XPS spectra after +20 V are shown in red and after –20 V are shown in blue. The peaks are assigned and the oxidation states are indicated above.

In order to understand the underlying mechanism of this voltage-induced change in the magnetic properties, we investigated the surface oxidation states using XPS. Fig. 4 shows the XPS spectra for Cu (left) and Fe (right), both for the as-sputtered (top) and nanoporous (bottom) films. XPS spectra were taken for the same film in the initial state (0 V, black lines), after +20 V was applied (red lines) and then after –20 V was applied (blue lines). The positive voltage was applied first in order to keep consistency with the magnetic studies presented above. The Cu spectra for the as-sputtered film at 0 V shows the presence of both Cu^0^ and Cu^2+^ peaks, most easily seen at 932.5 eV (Cu^0^) and 934.4 eV (Cu^2+^). After the application of +20 V (red line), there is a large increase in Cu^2+^ and a relative decrease in Cu^0^ meaning that the surface Cu was oxidized. Then, after the application of –20 V, the Cu^2+^ peak is almost gone, leaving mostly Cu^0^, which means the Cu was reduced. The same trends are observed in the porous film (bottom), although the dealloyed film is more oxidized than in the as-sputtered film. Please note that the peaks at 943 eV and 963 eV are satellite peaks from the Cu^2+^ signal and therefore are not evaluated closely as the main Cu^2+^ peak provides more valuable information.^43^ Looking at the Fe spectra (right side), for all voltages there are clear Fe^3+^ peaks and virtually no Fe^0^ peaks indicating that the Fe atoms on the surface are always oxidized, even after applying negative voltages. The Fe spectra for the porous sample is relatively weak and this is attributed to the low amount of Fe (compared to the as sputtered film) and the addition of residual solvent molecules on the very top surface which somewhat screen the measurement. The spectra for O was also measured and those results are presented in Fig. S2.† We find that the oxygen is in a mixture of metal oxides, metal hydroxides and a small amount of adsorbed water (the latter coming from the air moisture before the sample was placed in the vacuum chamber of XPS). The surface composition of the films was determined by XPS to be Fe_48_Cu_25_O_27_ for the as sputtered film and Fe_23_Cu_58_O_19_ for the porous film. Upon the application of +20 V, the surface oxygen content increases from 27 at% to 38 at% for the as sputtered film and 19 at% to 60 at% for the porous film. When –20 V is applied, the oxygen content decreases to 33 at% for the as sputtered film and 39 at% for the porous film. Please note that the values determined here are representative of the top few nanometers only as XPS is surface sensitive.

**Fig. 5:**
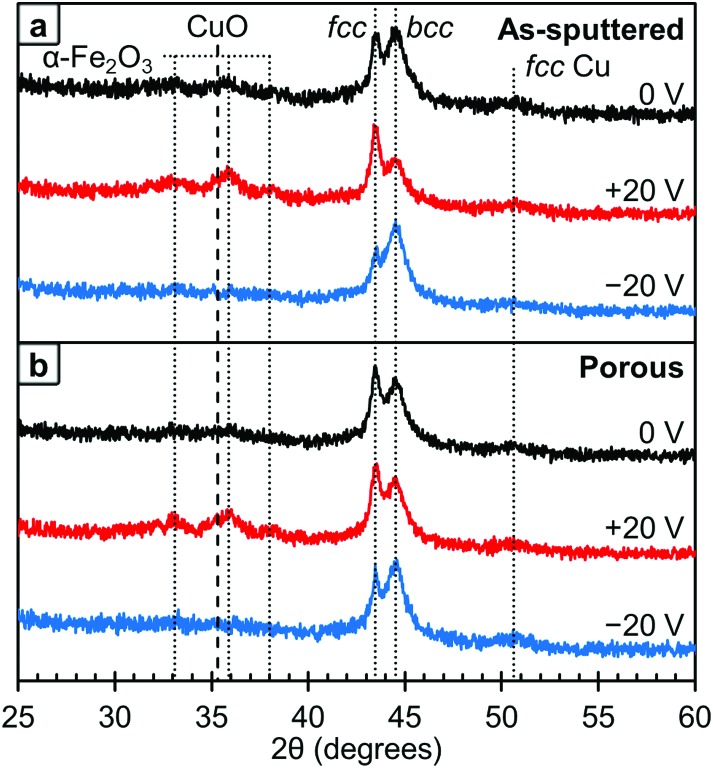
XRD patterns for the as-sputtered film (top) and the nanoporous film (bottom). The initial state (0 V) is shown in black, after +20 V is shown in red and after –20 V is shown in blue. The metallic peaks corresponding to the *fcc* and *bcc* phases, as well as the visible oxide peaks are labeled and assigned above.

The crystallographic structure of the films was probed using XRD and these results are presented in Fig. 5. As with the XPS, the data at different voltages was collected using the same film in the initial state (0 V, black lines), after +20 V was applied (red lines) and then after –20 V was applied (blue lines). The area under the *fcc* and *bcc* peaks were determined and used to estimate the relative ratio of the phases. We would like to note that this method is not perfect as any texturing *etc*. could affect the peak areas. Looking first at the as-sputtered film (Fig. 5a), there is a mixture of the *fcc* and *bcc* structures with a higher fraction of *bcc* (73 vol%) than *fcc* (27 vol%) as determined by peak area. After +20 V was applied, the ratio between *fcc* and *bcc* changes and an increase of the *fcc* fraction (61 vol%) is observed. Conversely, upon the application of –20 V, this change is reversed and the amount of *fcc* decreases to 13 vol% which is below the initial state. Furthermore, in the 0 V diffraction pattern, oxide peaks corresponding to Fe_2_O_3_ and CuO are barely observable. The XPS results show that there is some surface oxide present, so this suggests that the surface oxides are from a thin passivation layer that is approaching the detection limit of the XRD and, hence, almost at noise level. However, after +20 V is applied, these oxide peaks become better resolved suggesting that the films are becoming more oxidized. When –20 V is applied, the oxide peaks are undetectable. These results are consistent with the XPS results shown in Fig. 4. Between the XRD and XPS results, it appears that oxygen is leaving the films and therefore must be going into the electrolyte. While we are unsure of exactly what species of oxygen is formed, there are a number of possible soluble species including OH^–^, Na·O_2_, O_2_ or oxygen stabilized by a degradation product of the solvent, propylene carbonate.^44^ Looking at the crystal structure, in the porous films (Fig. 5b), the initial state is also a mixture of the *fcc* and *bcc* structures with a higher *fcc* fraction (37 vol%) than in the as-sputtered film (27 vol%). After +20 V was applied, the *fcc* fraction increases slightly to 42 vol% which is smaller than the change observed in the as-sputtered film which went from 27 vol% to 61 vol% *fcc* fraction. However, after –20 V was applied, the *bcc* fraction increased to 81 vol%. As with the as-sputtered film, the oxide peaks are only clearly detected after +20 V was applied.

We believe that the change in magnetic properties as a function of voltage observed here is primarily a magneto-ionic phenomenon. The XPS results (Fig. 4) clearly show the oxidation of Cu at +20 V and its subsequent reduction at –20 V. This is in agreement with the XRD patterns (Fig. 5) in which oxide peaks are much more prominent after the application of a positive voltage. We believe that this oxidation and reduction is the driving force behind the observed phase transformations between the *fcc* and *bcc* phases. Since Cu is the more noble metal, it is expected that the Fe would oxidize first. This is consistent with the XPS results, which show that on the surface, the Fe is entirely oxidized under all conditions, but some of the Cu remains metallic. When a positive voltage is applied and the films further oxidize, the Fe is expected to oxidize first which would effectively pull it out of the alloy. This then creates a more Cu-rich alloy which promotes the *fcc* structure and leads to a lower *M*_S_. This is consistent with the XRD results (Fig. 5) which show an increase in the *fcc* fraction when +20 V is applied, especially in the as-sputtered film (Fig. 5a). Furthermore, there is an observed decrease in *H*_C_, which would be expected from Fe in the *fcc* phase. When the films are reduced with a negative voltage, the Fe that had been previously oxidized by the positive voltage would then be reduced and reinserted into the alloy, effectively increasing the Fe content. This would in turn promote the *bcc* phase as well as a higher *M*_S_. Indeed, we do observe an increase in the *bcc* phase when a negative voltage is applied, both for the as-sputtered and nanoporous films (Fig. 5). This is consistent with the observed increase in *H*_C_ upon the application of negative voltages since Fe in the *bcc* phase should have a higher *H*_C_. The reason behind the increase in the *fcc* fraction upon oxidation being more pronounced in the as-sputtered than in the nanoporous film is, we suspect, due their differences in composition. Namely, the nanoporous film is more Cu-rich initially (Fe_34_Cu_66_) and has a higher *fcc* fraction initially (37 vol%), so the oxidation of Fe in that system cannot promote much further the *fcc* phase. The as-sputtered film, however, is more Fe-rich (Fe_58_Cu_42_) and has a higher *bcc* fraction (73 vol%), so the oxidation of Fe has a larger impact and the possible increase in the *fcc* fraction is larger.

In addition to the role of the *fcc*-to-*bcc* volume fraction, oxidation/reduction itself could also contribute, to some extent, to the observed changes in the magnetic properties (Fig. 3). The oxide peaks from the XRD patterns of the 0 V state correspond to α-Fe_2_O_3_, which is antiferromagnetic, and CuO, which is paramagnetic at room temperature. When positive voltages are applied, the amounts of α-Fe_2_O_3_ and CuO are increased, which could contribute to the decrease in *M*_S_. The oxygen in the sample is expected to come from the sample being stored in air (surface oxide passivation layer) although further oxygen is expected to be supplied in small amounts by absorbed water from the atmosphere, dissolved O_2_ in solution and possibly to a lesser extent, the OH^–^ ions in the electrolyte. Then, as observed by XRD and XPS, the application of a negative voltage reduces the films to some extent, which can cause the increase of the *M*_S_ and *H*_C_. We expect that the negative voltage does not reduce all of the oxide, which is why *M*_S_ does not fully recover back to its initial values. This irreversibility is likely largely a kinetic effect, since ionic diffusion is a relatively slow process and it can be asymmetric for positive and negative voltages. In order to assess this, we measured a new hysteresis loop 14 h after the measurement at –20 V had been made. The data from these loops are included in Fig. S1† for both the as-sputtered and porous films. We found that the as-sputtered film recovered 7% of its original *M*_S_ and the porous film recovered 13%. Similarly, the as-sputtered film recovered 50% of its original *H*_C_ and the porous film recovered 66%. This suggests that, at least in part, *M*_S_ and *H*_C_ do not immediately fully recover after the application of a negative voltage, due to kinetic limitations.

It is difficult to determine how much of the observed changes are directly due the oxidation/reduction and how much is due to the phase transformation. We suspect that the changes in *M*_S_ might be primarily influenced by the oxidation/reduction process. However, the changes in *H*_C_ are more difficult to interpret since they are likely determined by the interplay of the oxidation/reduction causing changes in local alloy composition and the phase transformations. We conjecture that for the porous sample, the change in alloy composition (relatively more Cu-rich after +20 V and more Fe-rich after –20 V) is the dominant factor in determining the *H*_C_. This explains why at positive voltages the *H*_C_ decreases and then increases for negative voltages. The as sputtered film on the other hand could be dominated more by microstructural factors including dipolar interactions between ferromagnetic grains or crystal structure, which may cancel out the effects of composition.

It is also worth noting that these redox processes occur in a controlled manner that preserves the morphology of both the as-sputtered and nanoporous films. This is because propylene carbonate does not participate in the redox process. We would expect that if a water-based electrolyte had been used, the films would dissolve at +20 V and the application of –20 V would cause the quick re-deposition of Cu and Fe on the substrate resulting in a loss of film integrity. Here, the films remain in the solid state during the entire process and the nanoporosity of the dealloyed films remains intact.

In addition to the magneto-ionic effects already discussed, pure magnetoelectric effects could also be playing some role on the observed changes in magnetic properties. Pure magnetoelectric effects are changes in the magnetic properties directly induced by the applied voltage (electrostatic charge accumulation), without any redox processes involved. In metals, these effects are often only observed in ultrathin films because the electric field is screened by the metal within the so-called Thomas–Fermi screening length (typically of less than 1 nm thickness).^45^ Therefore, pure electric field effects can affect only the top few nanometers. Pure magnetoelectric effects have also been observed in nanoporous films including metals with sufficiently small element (ligament or pore wall) sizes^46^ as well as porous metal oxides.^47^,^48^ Note that electric fields can penetrate metal oxides. For these reasons, we believe that pure magnetoelectric effects did not greatly affect the as-sputtered film, as it was too thick (∼200 nm). However, it is possible that pure magnetoelectric effects will contribute to some extent to the large magnetoelectric changes observed in the nanoporous dealloyed films.

## Conclusions

Here we have reported on the control of magnetism by applied voltage in Fe–Cu thin films. We investigated both an as-sputtered film (with nanoparticulate morphology) as well as a sputtered film made nanoporous *via* electrochemical dealloying. We have shown that the application of a positive voltage drastically decreases both the saturation magnetization (*M*_S_) and coercivity (*H*_C_) and that negative voltage causes an increase in both *M*_S_ and *H*_C_. Detailed studies by XPS and XRD reveal that the applied voltages cause oxidation and reduction of the samples, which indicates that magnetoionic effects play a role in the changes of magnetic properties. However, unlike other magnetoionic studies, here the oxidation/reduction of the samples induces reversible changes in the crystallographic structure (*i.e.*, the ratio of *fcc* to *bcc*), which in turn affects the magnetic properties. Overall, our results show that an applied voltage can be used to significantly influence the magnetic properties of Fe–Cu thin films. Similar results could be obtained in other system with co-existing crystal structures, hence paving the way for energy-efficient magnetic actuation and new types of sustainable spintronic devices.

## Conflicts of interest

The authors declare no competing financial interests.

## Supplementary Material

Supplementary information
