# Supplementary material for: Large magnetoelectric effects mediated by electric-field-driven nanoscale phase transformations in sputtered (nanoparticulate) and electrochemically dealloyed (nanoporous) Fe–Cu films
Source: Nanoscale. 2018 Jul 13;10(30):14570–8. doi: 10.1039/c8nr03924k (PMC6244191; doi:10.1039/c8nr03924k)
Supplement: Supplementary file 1 [file NR-010-C8NR03924K-s001.pdf]

Electronic Supplementary Material (ESI) for Nanoscale.
This journal is © The Royal Society of Chemistry 2018

Large magnetoelectric effects mediated by electric-field-driven nanoscale phase transformations in sputtered (nanoparticulate) and electrochemically dealloyed (nanoporous) Fe-Cu films

Shauna Robbennolt,[a,†] Alberto Quintana,[a] Eva Pellicer,[a] Jordi Sort[a,b,‡]

[a] Departament de Física, Universitat Autònoma de Barcelona, E-08193 Bellaterra, Spain
[b] Institució Catalana de Recerca i Estudis Avançats (ICREA), Pg. Lluís Companys 23, E-08010 Barcelona, Spain

Corresponding Authors:
† Shauna Robbennolt (shaunaaryn.robbennolt@uab.cat)
‡ Jordi Sort (jordi.sort@uab.cat)

1. Calculation of porosity in the films

Calculation of porosity for the as-sputtered sample ($Fe_{58}Cu_{42}$)

Unit cell length (a=b=c) = 361 pm
Sample density: 8.32 g/mL
Sample mass: 20.9 µg
Sample volume: 2.51 µL
Theoretical volume: 3 mm × 5 mm × 200 nm = 3 µL

Porosity: 84%

Calculation of porosity for the dealloyed sample ($Fe_{34}Cu_{66}$)

Unit cell length (a=b=c) = 361 pm
Sample density: 8.58 g/mL
Sample mass: 10.8 µg
Sample volume: 1.26 µL
Theoretical volume: 3 mm × 5 mm × 200 nm = 3 µL

Porosity: 58%

The unit cell length was determined from the *d*-spacing of the XRD peaks and then used in conjunction with the ratio of Fe:Cu and their molar masses to determine the sample density. The total mass of the sample (determined by ICP analysis) was divided by that density to give a total volume. Separately, the theoretical volume of the sample was calculated from the sample dimensions and film thickness. The volume fraction was then found by dividing the volume determined by XRD by the theoretical volume to be 42% making the porosity 58 vol%.

2. Further magnetoelectric data

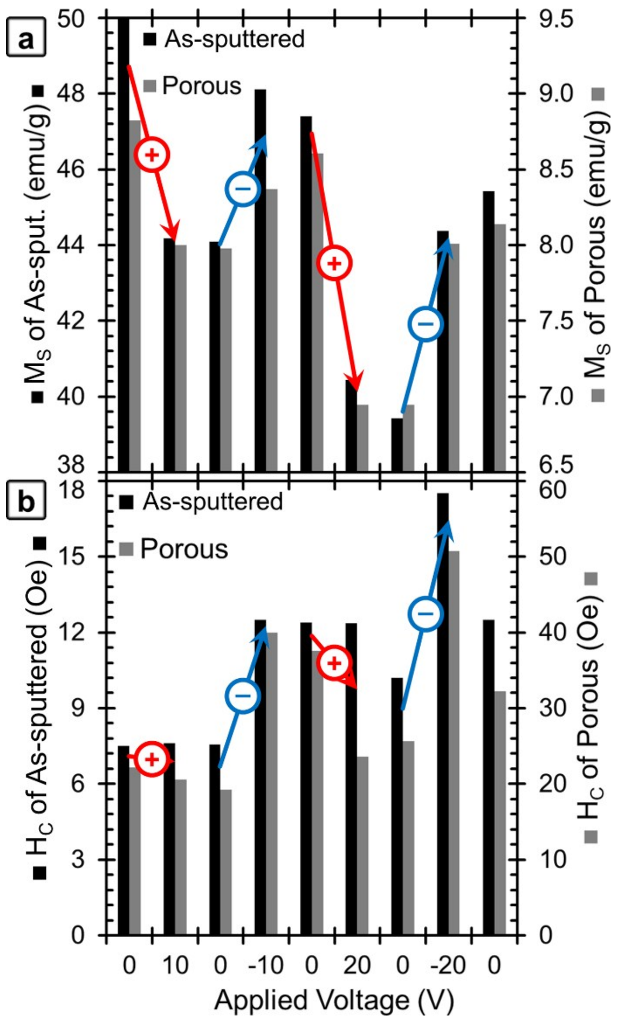

Figure S1 A graphical presentation of the dependence of (a) M_S and (b) H_C on the applied voltage for the as-sputtered (black, left axis) and porous (grey, right axis) samples. The data in presented in **Figure 3** is included along with all of the 0 V measurements between the other voltages. The voltages are listed in chronological order. For all but the last 0 V data, the samples were soaked at the voltage for 40 min and then the voltage continued to be applied during the 20 min measurement. For the final 0 V data however, the sample was left at 0 V for 14 h (overnight) before the measurement began in order to evaluate the role of kinetics.

3. Further XPS data

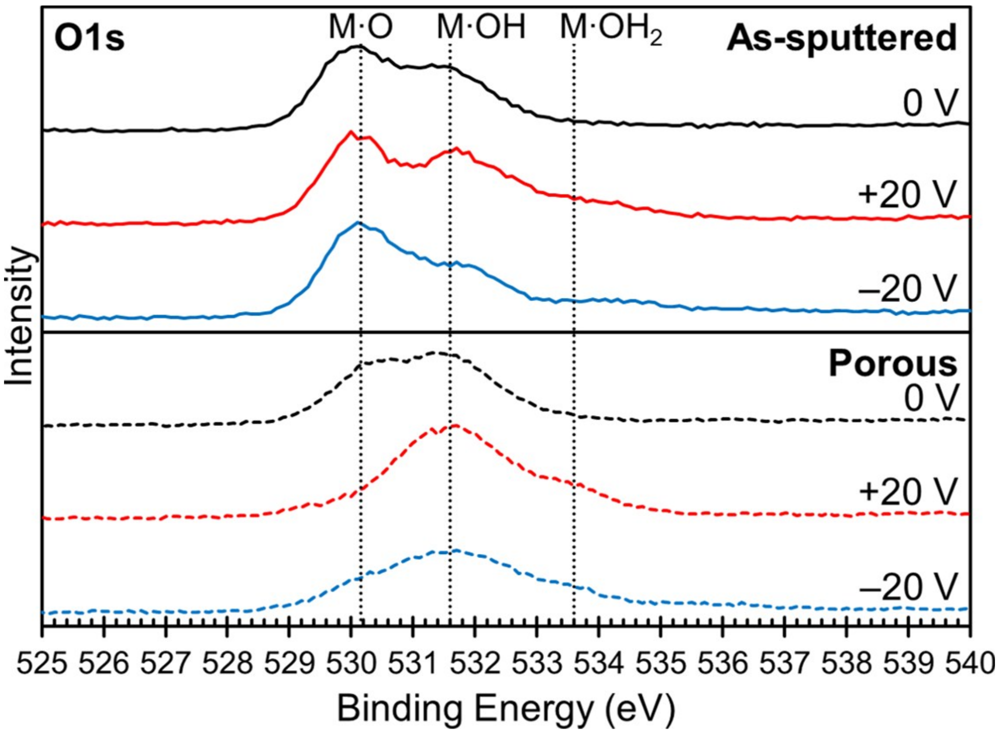

Figure S2. XPS elemental spectra of Oxygen for the as-sputtered film (top) and the nanoporous film (bottom). The initial state (0 V) is shown in black, while the XPS spectra after +20 V are shown in red and after -20 V are shown in blue. The peaks are assigned based on the type of complex they form with the metal. Metal oxide (M·O), metal hydroxide (M·OH), and water adsorbed to metal (M·OH_2). No molecular oxygen was detected.
